# Supplementary material for: Targeting CDK2 overcomes melanoma resistance against BRAF and Hsp90 inhibitors
Source: Mol Syst Biol. 2018 Mar 5;14(3):e7858. doi: 10.15252/msb.20177858 (PMC5836539; doi:10.15252/msb.20177858)
Supplement: Supplementary file 1 — Appendix [file MSB-14-e7858-s001.pdf]

APPENDIX

Table Of Content

Supplementary Materials and methods **3-18**

- Chemicals and reagents 3
- Cell lines and culture conditions 3
- Sample preparation for TPP 4-5
- Basic reverse phase fractionation 5
- TPP 5-6
- Data and software availability for TPP 6
- Phospho-TPP 6-7
- Data and software availability for phospho-TPP 7-8
- Sample preparation for protein expression levels analysis 8-9
- Protein expression levels analysis 9
- Phosphopeptide enrichment 9-10
- LC/MS/MS analysis 10
- Data processing 10-11
- Data and software availability 11
- Statistics 11-12
- Reactome analysis 12
- KEA2 and Enrichr 12
- Gene ontology enrichment analysis by GO Gorilla 12
- Concentration-response curves 13
- Isobolograms 13
- Proliferation assay (MTS) 13-14
- Flow cytometry based immunostaining and apoptosis/necrosis analysis 14

- Western blot 14
- Plasmid generation and creation of inducible stable cell lines 14-15
- MITF and CDK2 correlation analysis in TCGA 15-16
- References 16-18

Supplementary Materials and methods

Chemicals and reagents

The drugs employed in this study were: dabrafenib (Apexbio, B1407-50); XL888 (Apexbio, A4388-25); Fludarabine (Selleckchem, S1491); CHIR-99021 HCl (CT99021) (Selleckchem, S2924); Palbociclib (Apexbio, A8316); LDC000067 (Apexbio, B4754-10); Ro 3306 (Apexbio, A8885-10); roscovitine (Apexbio, A1723-10); K03861 (Selleckchem, S8100); CHIR-99021 (Selleckchem, S2924); dinaciclib (Selleckchem, S2768); FRAX597 (Selleckchem, S7271); PF-3758309 (Selleckchem, S7094); AUY922 (Selleckchem, S1069); BIIB021 (Selleckchem, S1175); novobiocin (Selleckchem, S2492); 17-DMAG (Selleckchem, S1142). All the drugs were dissolved in DMSO (Sigma D2650).

Cell lines and culture conditions

SK-Mel 24, SK-Mel 28, A375, A375DR1 (dabrafenib resistant; 1 μM), MNT-1 (kindly provided by Dr Pier Giorgio Natali, Istituto Regina Elena, Rome, Italy), MNT-1-DR100 (dabrafenib resistant, 100 nM) cells were grown in Gibco Medium Essential medium (MEM; ThermoFisher Scientific) supplemented with 10% FBS (15% for SK-Mel 24), 1% non-essential amino acids, 1% sodium pyruvate, 1% penicillin and streptomycin. ESTDAB37 and ESTDAB102 (received from The European Searchable Tumour Line Database (ESTDAB)), SKMEL2, M026.X1.CL, M026R.X1.CL, M029.X1.CL and M029R.X1.CL (post relapse, resistant to BRAF inhibitor treatment) (Kemper et al, 2016;Possik et al, 2014) were grown in Gibco RPMI 1640 (ThermoFisher Scientific) supplemented with 10% FBS, 1% non-essential amino acids, 1% sodium pyruvate, 1% penicillin streptomycin.

Sample preparation for TPP

Intact cells - SK-Mel 24 and SK-Mel 28 were incubated in the presence of DMSO (control) or drug (100 μM) for 2 h. Cells were harvested, resuspended in 1.1 ml of HBBS (Gibco) supplemented with 1 mM sodium orthovanadate, 1 tablet of Complete mini EDTA-free mixture (Roche Applied Science) and one tablet of PhosSTOP phosphatase inhibitor mixture per 10 ml of lysis buffer (Roche Applied Science). The cell suspension per cell line (SK-Mel 24 and SK-Mel 28) per condition (+/- drug) was divided into ten aliquots of 100 µl and transferred into 0.2 ml PCR tubes. TPP was performed as previously described (Franken et al, 2015). Briefly, each tube was heated individually at the different temperatures for 3 min in a thermal cycler (Applied Biosystems (Foster City, CA)/Life Technologies) followed by cooling for 3 min at room temperature. Cell were lysed by freeze&thaw cycles and the lysates were centrifuged at 20,000 x g for 30 min at 4°C to separate the soluble fractions from precipitates. The supernatants were transferred to new 0.2 ml microtubes.

Lysate - SK-Mel 24 and SK-Mel 28 were lysed in lysis buffer (HBBS (Gibco) supplemented with 1 mM sodium orthovanadate, 1 tablet of Complete mini EDTA-free mixture (Roche Applied Science) and one tablet of PhosSTOP phosphatase inhibitor mixture per 10 ml of lysis buffer (Roche Applied Science)) by freeze&thaw and the lysates were centrifuged at 20,000 x g for 30 min at 4°C to separate the soluble fractions from precipitates. Each cell line's supernatant were incubated with either DMSO or 100 μM drug at room temperature for 15 min. The cell suspension per cell line (SK-Mel 24 and SK-Mel 28) per condition (+/- drug) was divided into ten aliquots of 100 µl and transferred into 0.2 ml PCR tubes. TPP was performed as previously described (Franken et al, 2015).

Each supernatant for cell line (SK-Mel 24 and SK-Mel 28); condition (+/- drug); and experiment (intact cells cell/lysate) was reduced by 2 mM DTT at room temperature for 1 h;

alkylated by 4 mM chloroacetamide for 30 min at room temperature at the dark. A first enzymatic digestion was performed using Lys-C (1:75 w/w) at 37°C overnight; a second enzymatic digestion was performed using Trypsin (1:75 w/w) at 37°C overnight. One hundred µg of each sample was labelled by TMT10plex according to the manufacturer instructions. Samples of each set (10 different temperatures) were mixed 1:1 (v/v) and cleaned by Strata ^{TM}-X-C 33 µm Polymeric Strong Cation (Phenomenex). The whole procedure was executed in two biological replicates.

Basic reverse phase fractionation

Two hundred µg of peptide mixture from each set were fractionated using a Waters XBridge BEH300 C18 3.5 µm 2.1×250 mm column on a Agilent 1200 series operating at 200 µl/min. Buffer A consisted of 20 mM NH_3, while buffer B of 80% ACN / 20 mM NH_3. The fractionation gradient was: 3-88% B in 63 min; 88% B for 15 min; and ramped to 100% B in 2.5 min; 100% B for 13.5 min. Fractions were collected into a polypropylene V-96 well microtiterplates (Microplate, 96 well PP, V-Bottom; Grainer BIO-ONE). At 97 min, fraction collection was halted, and the gradient was held at 3% B for 20 min. The total number of concatenated fractions was set to 12. Each plate was dried at room temperature using a Speed Vac (SPD 111V, Thermo). Plates were stored at -20°C till LC/MS/MS analysis.

TPP

Each sample was analyzed on a HF Q-Exactive Orbitrap (Thermo Fisher, Germany) connected to a Dionex UHPLC system (Thermo Fisher Scientific, Germany). The UHPLC was equipped with a trap column (Acclaim PepMap 100, 75 µm x 2 cm, nanoviper, C_{18}, 3 µm, 100 Å; Thermo Fisher Scientific, Germany) and an analytical column (PepMap RSLC C_{18}, 2 µm, 100 Å, 75 µm x 50 cm; Thermo Fisher Scientific, Germany). Mobile-phase buffers for

nLC separation consisted of 0.1% FA in water (solvent A) and 80% ACN/0.1% FA (solvent B). The peptides were eluted during a 2 h gradient and directly sprayed into the mass spectrometer. The flow rate was set at 250 nl/min, and the LC gradient was as follows: 3-6% solvent B within 3 min, 6-35% solvent B within 117 min, 35–47% solvent B within 5 min, 47-100% solvent B within 5 min and 100% B for 8 min and 1% solvent B for 5 min. Nano spray was achieved with an applied voltage of 1.8 kV. The mass spectrometer was programmed in a data-dependent acquisition mode (top 10 most intense peaks) and was configured to perform a Fourier transform survey scan from 370 to 1,600 *m/z* (resolution 60,000), AGC target 3 e6, maximum injection time 250 ms. MS2 scans were acquired on the 10 most-abundant MS1 ions of charge state 2–7 using a Quadrupole isolation window of 1 m/z for HCD fragmentation. Collision energy was set at 34%; resolution = 30 000; AGC target 2 e5, maximum injection time 200 ms; dynamic exclusion 15 s.

Data and software availability for TPP

The raw data were analyzed as previously described (Franken et al, 2015). Mascot was used to search the MS/MS data against the UniProt *Homo sapiens* database (containing canonical and isoforms_42144 entries downloaded on 21st March 2016). The data have been deposited to the ProteomeXchange Consortium (http://proteomecentral.proteomexchange.org) via the PRIDE partner repository (Vizcaino et al, 2013) with the dataset identifier PXD005508. PX reviewer account: username: reviewer17590@ebi.ac.uk; password: pdjwoM08.

Phospho-TPP

For TiO_2 phosphopeptide enrichment ~600 μg of TMT labeled sample for each cell line (SK-Mel 24 and SK-Mel 28); condition (+/- drug); and experiment layout (intact cells/lysate) were used. The enrichment was executed as previously described (Matheron et al, 2014). The raw

data were analyzed by MaxQuant (version 1.5.3.30) (Cox & Mann, 2008) and Andromeda (Cox et al, 2011) was used to search the MS/MS data against the UniProt *Homo sapiens* database (containing canonical and isoforms_42144 entries downloaded on 21st March 2016) complemented with a list of common contaminants and concatenated with the reversed version of all sequences. TMT10plex was chosen as quantification platform. Trypsin/P was chosen as cleavage specificity allowing two missed cleavages. Carbamidomethylation (C) was set as a fixed modification, while oxidation (M) and phosphorylation of STY were used as variable modification. The database search was performed with a mass deviation of the precursor ion of up to 4.5 ppm (main search). Data filtering was carried out using the following parameters: peptide and protein FDRs were set to 1%, minimum peptide length was set to 7 and Andromeda minimum score for modified peptides was set to 40. Match between runs was enabled. The reverse and common contaminant hits were removed from MaxQuant output.

Data and software availability for phospho-TPP

The mass spectrometry proteomics data have been deposited to the ProteomeXchange Consortium (http://proteomecentral.proteomexchange.org) via the PRIDE partner repository (Vizcaino et al, 2013) with the dataset identifier PXD005547. PX reviewer account: username: reviewer54934@ebi.ac.uk; password: FO00iPQk.

The data were analyzed using an in-house R-package using the similar criteria that were previously described (Savitski et al, 2014). The raw data was analyzed as using similar workflow to one previously described (Franken et al, 2015) except that the each detected peptide was fit and analysed individually instead of at a protein level. The same qualifiers for normalization and curve inclusion in analysis were used. P-values were then calculated as previously described and then the following criteria were developed to rank the resulting

shifts: 1) the thermal shift must be in the same direction for both replicates (negative or positive), 2) the difference between melting point of the two vehicle curves must be smaller than the thermal shifts from both replicates, 3) the minimum shift must be > 0.7 * the maximum shift, 4) the slope of the curve must be greater than -0.06, and 5) the p-value must be greater than 0.2 in both replicates. The resulting hit list was then manually inspected. MaxQuant (version 1.5.3.30) (Cox & Mann, 2008) and Andromeda (Cox et al, 2011) were used to search the MS/MS data against the UniProt *Homo sapiens* database (containing canonical and isoforms_42144 entries downloaded on 21st March 2016) complemented with a list of common contaminants and concatenated with the reversed version of all sequences. Trypsin/P was chosen as cleavage specificity allowing two missed cleavages. Carbamidomethylation (C) was set as a fixed modification, while oxidation (M) and phosphorylation of STY were used as variable modification. Data filtering was carried out using the following parameters: peptide and protein FDRs were set to 1%, minimum peptide length was set to 7 and Andromeda minimum score for modified peptides was set to 40. The resulting hit list was then manually inspected.

The script is available as 'Computer Scrips EV1'.

Sample preparation for protein expression levels analysis

SK-Mel 24 and SK-Mel 28 were grown in four different conditions: in the presence of DMSO (control); 1 µM dabrafenib; 200 nM XL888 ; and 1 µM dabrafenib plus 200 nM XL888 each for 48 h. Cell pellets were harvested and resuspended in lysis buffer (8M Urea, 100 mM triethylammonium bicarbonate pH 8.5, 1 mM sodium orthovanadate, 1 tablet of Complete mini EDTA-free mixture (Roche Applied Science) and one tablet of PhosSTOP phosphatase inhibitor mixture per 10 ml of lysis buffer (Roche Applied Science)). Cells were then lysed by 10 rapid passages through a 23-gauge hypodermic syringe needle and by sonication on ice.

After centrifugation (20,000 × g 30 min at 4°C), the protein concentration was determined by Bradford assay (Pierce). Proteins were reduced with 2 mM DTT at room temperature for 1 h, alkylated with 4 mM chloroacetamide at room temperature for 30 min in the dark. A first enzymatic digestion step was performed using Lys-C at 37°C for 4 h (enzyme/substrate ratio 1:50). Moreover, the sample was digested overnight at 37°C with Trypsin (enzyme/substrate ratio 1:50). Peptides were desalted by reverse phase using Waters Sep-Pak 1 cc (50 mg) cartridges (WAT054960; Waters, Milford, MA). The resin was rinsed with ACN and then equilibrated with 0.6% acetic acid. The samples were loaded and washed with 0.6% acetic acid, eluted with 80% ACN/0.6% acetic acid.

Protein expression levels analysis

A label-free approach was used for the analysis of protein expression levels: 2 µg of each sample was analysed in three biological and (at least) two technical replicates on a Fusion Orbitrap (Thermo Fisher, Germany).

Phosphopeptide enrichment

A phosphopeptide enrichment by TiO_2 was performed on each sample in three biological and (at least) two technical replicates. TiO_2 material was prepared used as previously described (Matheron et al, 2014). Briefly, the enrichment procedure was as follows: the TiO_2 material was pre-equilibrated two times with 50 µl of loading buffer (80% ACN, 6% trifluoroacetic acid (TFA)). Next, 250 µg of each sample was resuspended in 50 µl of loading buffer and loaded onto the equilibrated GELoader tips. Then, the TiO_2 material was washed with 50 µl wash buffer A (50% ACN, 0.5% TFA, 200 mM NaCl) and subsequently with 50 µl wash buffer B (50% ACN, 0.1% TFA). Bound peptides were first eluted by 20 µl of 10% ammonia into 30 µl of 10% FA. Finally, the remained peptides were eluted with 2 µl of 80% ACN, 2%

FA. The collected elute was further acidified by adding 3 µl of 100% FA, dried down and stored at -80°C till the LC/MS/MS analysis.

LC/MS/MS analysis

Each sample was analyzed on a Fusion Orbitrap (Thermo Fisher, Germany) connected to a Dionex UHPLC system (Thermo Fisher Scientific, Germany). The UHPLC was equipped with a trap column (Acclaim PepMap 100, 75 µm x 2 cm, nanoviper, C_{18}, 3 µm, 100 Å; Thermo Fisher Scientific, Germany) and an analytical column (PepMap RSLC C_{18}, 2 µm, 100 Å, 75 µm x 50 cm; Thermo Fisher Scientific, Germany). Mobile-phase buffers for nLC separation consisted of 0.1% FA in water (solvent A) and 80% ACN/0.1% FA (solvent B). The peptides were eluted during a 2 h gradient and directly sprayed into the mass spectrometer. The flow rate was set at 250 nl/min, and the LC gradient was as follows: 1-6% solvent B within 7 min, 6-37% solvent B within 113 min, 30–100% solvent B within 5 min, 100% solvent B for 8 min and 1% solvent B for 10 min. Nano spray was achieved with an applied voltage of 1.8 kV. The mass spectrometer was programmed in a data-dependent acquisition mode (top 10 most intense peaks) and was configured to perform a Fourier transform survey scan from 350 to 1,550 *m/z* (resolution 120,000), RF lens (%)=60, AGC target 2 e5, maximum injection time 50 ms, 1 microscan, 30 s as dynamic exclusion. MS2 scans were acquired on the 10 most-abundant MS1 ions of charge state 2–7 using a Quadrupole isolation window of 1.4 m/z for CID fragmentation. CID activation was set with a collision energy of 32%, ion trap Rapid scan rate, AGC target of 1 e4; and maximum injection time of 100 ms, 1 microscan, centroid.

Data processing

Label-free quantification was performed by analyzing the raw data by MaxQuant (version 1.5.3.30) (Cox & Mann, 2008). Andromeda (Cox et al, 2011) was used to search the MS/MS data against the UniProt *Homo sapiens* database (containing canonical and isoforms_42144 entries downloaded on 21st March 2016) complemented with a list of common contaminants and concatenated with the reversed version of all sequences. Trypsin/P was chosen as cleavage specificity allowing two missed cleavages. Carbamidomethylation (C) was set as a fixed modification, while oxidation (M) was used as variable modification. In addition, for the phosphosproteome analysis phosphorylation of STY Peptide identification was use as variable as well. The database search was performed with a mass deviation of the precursor ion of up to 4.5 ppm (main search), and the allowed fragment mass deviation was set to 0.5 Da for ITMS. Data filtering was carried out using the following parameters: peptide and protein FDRs were set to 1%, minimum peptide length was set to 7 and Andromeda minimum score for modified peptides was set to 40. Match between runs was enabled. The reverse and common contaminant hits were removed from MaxQuant output.

Data and software availability

The mass spectrometry proteomics and phosphoproteomics data have been deposited to the ProteomeXchange Consortium (http://proteomecentral.proteomexchange.org) via the PRIDE partner repository (Vizcaino et al, 2013) with the dataset identifier PXD005518. PX reviewer account: username: reviewer11573@ebi.ac.uk; password: NdWZLtSv.

Statistics

To perform a pairwise comparison and filter for those proteins/phosphopeptides that have a consistent abundance level over three biological replicates, we applied a two-sample *t*-test using Perseus 1.5.3.2 (Cox & Mann, 2008). Only those proteins that had a p-value < 0.05 and

an arbitrary cut-off ratio ≥ 1.5 or ≤ −1.5 fold changes were considered. Only phosphopeptides with a location probability ≥ 0.75 were considered for statistical analyses. In addition, using Perseus 1.5.3.2 a PCA analysis was performed comparing the two cell lines in the four different conditions.

Reactome analysis

The statistical significant protein entries and phosphopeptides were analyzed by Cytoscape 3.2 (Smoot et al, 2011) using Reactome (Haw et al, 2011).

KEA2 and Enrichr

The statistical significant phosphopeptides were analyzed by KEA2 (Lachmann & Ma'ayan, 2009) to predict kinases activity.

The statistical significant protein entries were analyzed by Enrichr (Kuleshov et al, 2016), using ChEA (Lachmann et al, 2010), to predict the transcription factor activity from the input data.

Gene ontology enrichment analysis by GO Gorilla

GO Gorilla was used to perform gene ontology (GO) (Eden et al, 2009). For the proteome analysis, the software was run using both target (significant regulated proteins with a fold change ≥ 1.5 or ≤ −1.5) and background list (the complete list of identified proteins), to calculate enrichment of biological processes across the target list. The same procedure was performed for the phosphoproteome. A Benjamini & Hochberg false discovery rate correction was applied to correct for multiple testing. Enriched biological processes with a *P*-value < 0.01 were considered.

Concentration-response curves

Compounds were serially diluted in DMSO with a BRAVO liquid handler and 300 nl was spotted into a 384-well microplate using an Echo555. 1000 cells/well were seeded in 384-well microplates in 20 µl with a multidrop and incubated for 24 hours at 37°C. The compound solutions were then dissolved in 30 µl media and 10 µl of the solution was transferred to the microplates containing cells using a BRAVO liquid handler. Plates were incubated for 48 or 72 h at 37°C and then 30 µl CellTiter-Glo diluted 1:3 with PBS was added using a multidrop. After 10 min incubation at room temperature the luminescence of each well was recorded on an EnVision (Perkin Elmer). The percent viability in each well was computed by comparing to the positive (6 µM staurosporine) and the negative (DMSO) controls. The graphs have been generated in GraphPad.

Isobolograms

Compounds were serially diluted in DMSO and then dissolved in media. 1000 cells/well were seeded in 384-well microplates in 20 µl with a multidrop and incubated for 24 h at 37°C. The compound solutions were then dissolved in media and 10 µl of each solution containing compound was transferred to the microplates with the plated cells. Plates were incubated for 72 h at 37°C and then 30 µl CellTiter-Glo diluted 1:3 with PBS was added using a multidrop. After 10 min incubation at room temperature the luminescence of each well was recorded on an EnVision (Perkin Elmer). The percent viability in each well was computed by comparing to the positive (6 µM staurosporine) and the negative (DMSO) controls.

Proliferation assay (MTS)

CellTiter 96 AQueous One Solution Cell Proliferation Assay (MTS) was purchased from Promega (Cat. no. G3582, Promega, Madison, WI, USA). The graphs have been generated in GraphPad.

Flow cytometry based immunostaining and apoptosis/necrosis analysis

To evaluate the presence of apoptosis/necrosis, we used annexin V-Fluos (cat. no. 11828681001 Roche) and propidium iodide and analyzed by NovoCyte flow cytometer (ACEA biosciences, Inc. San Diego, CA). Between 4 - 10x10^4 cells per well were cultured and treated with XL888 or dinaciclib for 48 - 72 h or transfected with siRNA 48 h prior to the single or combination treatment. Then the cells were collected and rinsed in PBS, pelleted, and re-suspended in incubation buffer (10 mmol/l HEPES/NaOH, pH 7.4, 140 mmol/l NaCl, 5 mmol/l $CaCl_2$) containing 1% annexin V and 1% propidium iodide (PI) for 10 min.

Western blot

To validate selected protein candidates, protein extracts from SK-Mel 24, SK-Mel 28, ESTDAB37, MNT1, M029R.X1.CL cell lines were analyzed by immunoblotting using NuPAGE Novex Bis-Tris Gel (Life Technologies, Carlsbad, CA, USA) and PVDF membranes (Thermo Scientific, Rockford, IL, USA), according to the manufacturer's standard protocol. The employed antibodies were: AKT1 (Cell Signaling Technology, 75692S); CDK2 (ThermoFisher, MA513725); CDC37 (ThermoFisher, PA129131); Hsp90α/β (Santa Cruz, sc-13119), MITF (ThermoFisher, MA514146); pERK1 (Y204) / pERK2 (Y187) (Abcam, ab47339); β-actin (Cell Signaling Technology, 3700S); phospho PAK4 (Ser474); (Cell Signaling Technology, 3241S); DCT (Atlas antibodies, HPA010800).

Plasmid generation and creation of inducible stable cell lines

Inducible shRNA-constructs were created by ligating annealed shRNA-coding oligonucleotides (see Supplementary Table 8) into an AgeI/EcoRI double-digested inducible shRNA vector as described previously (Eshtad et al, 2016). The constructs were validated using sequencing over the shRNA insertion area. The plasmids, together with a non-targeting shRNA plasmid (Eshtad et al, 2016), were packaged into lentiviral particles using a third generation lentiviral production system described previously (Dull et al, 1998), using $CaCl_2$-mediated transfection of HEK293T-cells, and the produced lentiviral particles were used to infect SK-Mel 28 cells together with 0.4 μg/ml hexadimethrine bromide which were then selected for successful integration with 1 μg/ml puromycin over five days.

MITF and CDK2 correlation analysis in TCGA

Correlation between MITF and CDK2 mRNA expression was evaluated for many different cancer types in both primary tumor samples and cancer cell lines. For the tumor sample analysis, transcriptomic data (RNA-Seq, TPM) was retrieved for 33 different cancer types from The Cancer Genome Atlas (TCGA) project via the UCSC Xena database (http://xena.ucsc.edu). For each cancer type, the Pearson linear correlation coefficient between MITF and CDK2 TPM values was calculated, including only tumor-associated samples (“Primary Solid Tumor”, “Recurrent Solid Tumor”, “Additional - New Primary”, “Metastatic”, “Additional Metastatic”, or “Primary Blood Derived Cancer - Peripheral Blood”).

To analyze MITF and CDK2 correlation in human cancer cell lines, processed RNA-seq data (TPM values) for 935 different cell types from the Cancer Cell Line Encyclopedia (CCLE) was retrieved from the Cancer Target Discovery and Development (CTD2) Network (https://ocg.cancer.gov/programs/ctd2/data-portal), established by the National Cancer Institute’s Office of Cancer Genomics. Cell lines were grouped by cancer type origin (e.g.,

skin cutaneous melanoma, breast invasive carcinoma, etc.), and the Pearson linear correlation coefficient between MITF and CDK2 TPM values was calculated for each group.

For both primary tumor and cell line analyses, the significance (p-value) associated with each calculated Pearson coefficient was calculated based on a two-tailed test with an alternative hypothesis of a non-zero correlation, and was adjusted (padj) for the false discovery rate using the Benjamini-Hochberg procedure.

References

Cox J, Mann M (2008) MaxQuant enables high peptide identification rates, individualized p.p.b.-range mass accuracies and proteome-wide protein quantification. *Nat Biotechnol* **26:**1367-1372.

Cox J, Neuhauser N, Michalski A, Scheltema RA, Olsen JV, Mann M (2011) Andromeda: a peptide search engine integrated into the MaxQuant environment. *J Proteome Res* **10:**1794-1805.

Dull T, Zufferey R, Kelly M, Mandel RJ, Nguyen M, Trono D, Naldini L (1998) A third-generation lentivirus vector with a conditional packaging system. *J Virol* **72:**8463-8471

Eden E, Navon R, Steinfeld I, Lipson D, Yakhini Z (2009) GOrilla: a tool for discovery and visualization of enriched GO terms in ranked gene lists. *BMC Bioinformatics* **10:**48-2105-10-48.

Eshtad S, Mavajian Z, Rudd SG, Visnes T, Bostrom J, Altun M, Helleday T (2016) hMYH and hMTH1 cooperate for survival in mismatch repair defective T-cell acute lymphoblastic leukemia. *Oncogenesis* **5:**e275.

Franken H, Mathieson T, Childs D, Sweetman GM, Werner T, Togel I, Doce C, Gade S, Bantscheff M, Drewes G, Reinhard FB, Huber W, Savitski MM (2015) Thermal proteome

profiling for unbiased identification of direct and indirect drug targets using multiplexed quantitative mass spectrometry. *Nat Protoc* **10:**1567-1593.

Haw R, Hermjakob H, D'Eustachio P, Stein L (2011) Reactome pathway analysis to enrich biological discovery in proteomics data sets. *Proteomics* **11:**3598-3613.

Kemper K, Krijgsman O, Kong X, Cornelissen-Steijger P, Shahrabi A, Weeber F, van der Velden DL, Bleijerveld OB, Kuilman T, Kluin RJ, Sun C, Voest EE, Ju YS, Schumacher TN, Altelaar AF, McDermott U, Adams DJ, Blank CU, Haanen JB, Peeper DS (2016) BRAF(V600E) Kinase Domain Duplication Identified in Therapy-Refractory Melanoma Patient-Derived Xenografts. *Cell Rep* **16:**263-277.

Kuleshov MV, Jones MR, Rouillard AD, Fernandez NF, Duan Q, Wang Z, Koplev S, Jenkins SL, Jagodnik KM, Lachmann A, McDermott MG, Monteiro CD, Gundersen GW, Ma'ayan A (2016) Enrichr: a comprehensive gene set enrichment analysis web server 2016 update. *Nucleic Acids Res* **44:**W90-7.

Lachmann A, Ma'ayan A (2009) KEA: kinase enrichment analysis. *Bioinformatics* **25:**684-686.

Lachmann A, Xu H, Krishnan J, Berger SI, Mazloom AR, Ma'ayan A (2010) ChEA: transcription factor regulation inferred from integrating genome-wide ChIP-X experiments. *Bioinformatics* **26:**2438-2444.

Matheron L, van den Toorn H, Heck AJ, Mohammed S (2014) Characterization of biases in phosphopeptide enrichment by Ti(4+)-immobilized metal affinity chromatography and TiO2 using a massive synthetic library and human cell digests. *Anal Chem* **86:**8312-8320.

Possik PA, Muller J, Gerlach C, Kenski JC, Huang X, Shahrabi A, Krijgsman O, Song JY, Smit MA, Gerritsen B, Lieftink C, Kemper K, Michaut M, Beijersbergen RL, Wessels L, Schumacher TN, Peeper DS (2014) Parallel in vivo and in vitro melanoma RNAi dropout

screens reveal synthetic lethality between hypoxia and DNA damage response inhibition. *Cell Rep* **9:**1375-1386.

Savitski MM, Reinhard FB, Franken H, Werner T, Savitski MF, Eberhard D, Martinez Molina D, Jafari R, Dovega RB, Klaeger S, Kuster B, Nordlund P, Bantscheff M, Drewes G (2014) Proteomics. Tracking cancer drugs in living cells by thermal profiling of the proteome. *Science* **346:**1255784.

Smoot ME, Ono K, Ruscheinski J, Wang PL, Ideker T (2011) Cytoscape 2.8: new features for data integration and network visualization. *Bioinformatics* **27:**431-432.

Vizcaino JA, Cote RG, Csordas A, Dianes JA, Fabregat A, Foster JM, Griss J, Alpi E, Birim M, Contell J, O'Kelly G, Schoenegger A, Ovelleiro D, Perez-Riverol Y, Reisinger F, Rios D, Wang R, Hermjakob H (2013) The PRoteomics IDEntifications (PRIDE) database and associated tools: status in 2013. *Nucleic Acids Res* **41:**D1063-9.
