# Supplementary material for: Targeting CDK2 overcomes melanoma resistance against BRAF and Hsp90 inhibitors
Source: Mol Syst Biol. 2018 Mar 5;14(3):e7858. doi: 10.15252/msb.20177858 (PMC5836539; doi:10.15252/msb.20177858)
Supplement: Supplementary file 2 — Expanded View Figures PDF [file MSB-14-e7858-s002.pdf]

Expanded View Figures

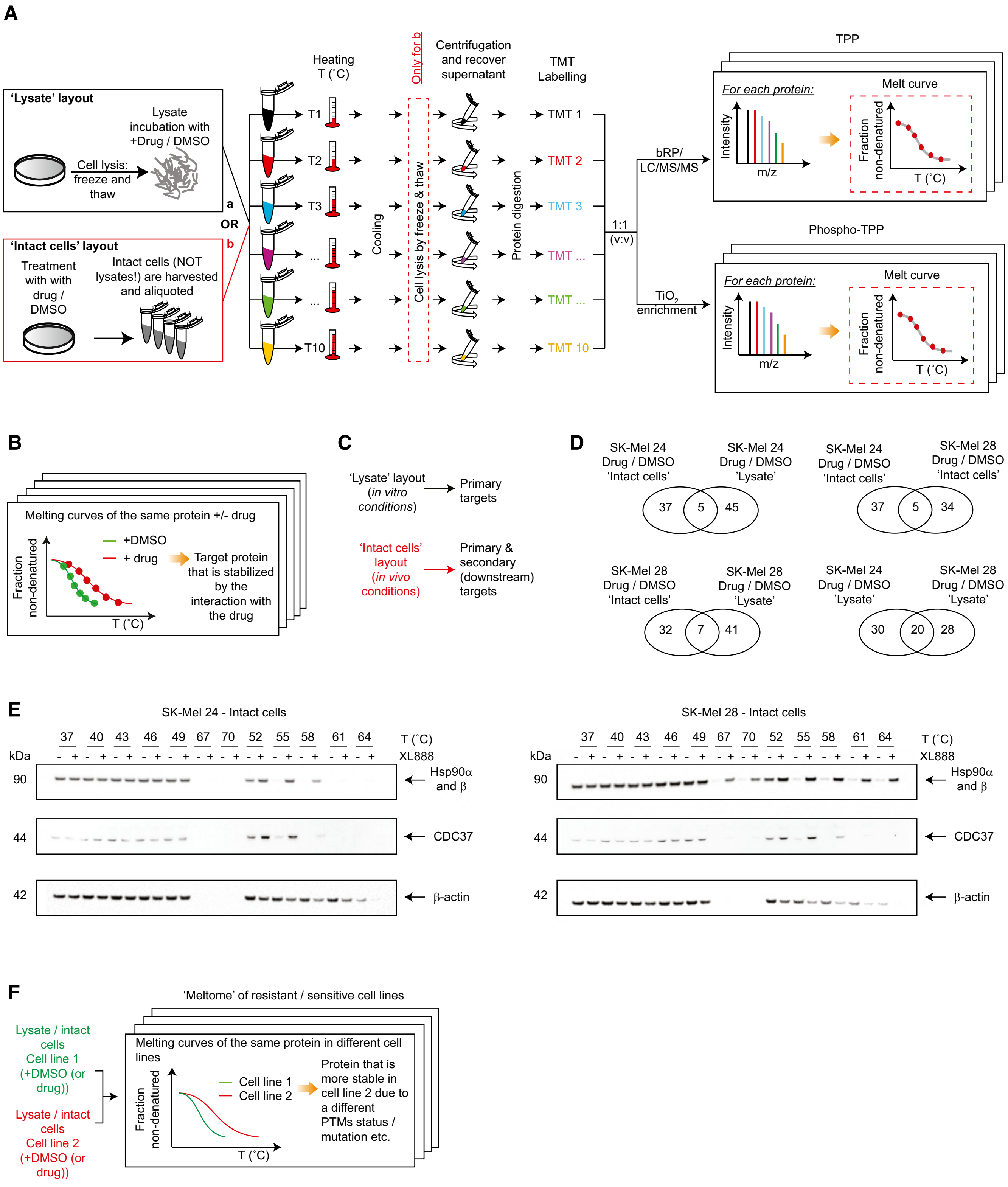

Figure EV1.

◀

Figure EV1. TPP platform employed in this study.

A Workflow of the TPP platforms ("lysate" and "intact cells") used to measure the proteome and phosphoproteome thermal stability upon drug treatment using a TMT approach.

B The comparison of the proteome thermal stability upon different conditions (+/− drug) enables to identify the drug targets.

C The two layouts "lysate" and "intact cells" provide complementary information regarding the nature of the targets (primary drug targets or secondary targets; Franken *et al*, 2015).

D Venn diagrams of the entries retrieved by the comparisons in different settings. Experiments were performed in two biological replicates.

E Validation of Hsp90 and CDC37 thermal shift by Western blot.

F The TPP workflow enables the comparison of the thermal stability of proteome and phosphoproteome of resistant versus sensitive cells to the Hsp90i XL888.

© 2018 The Authors

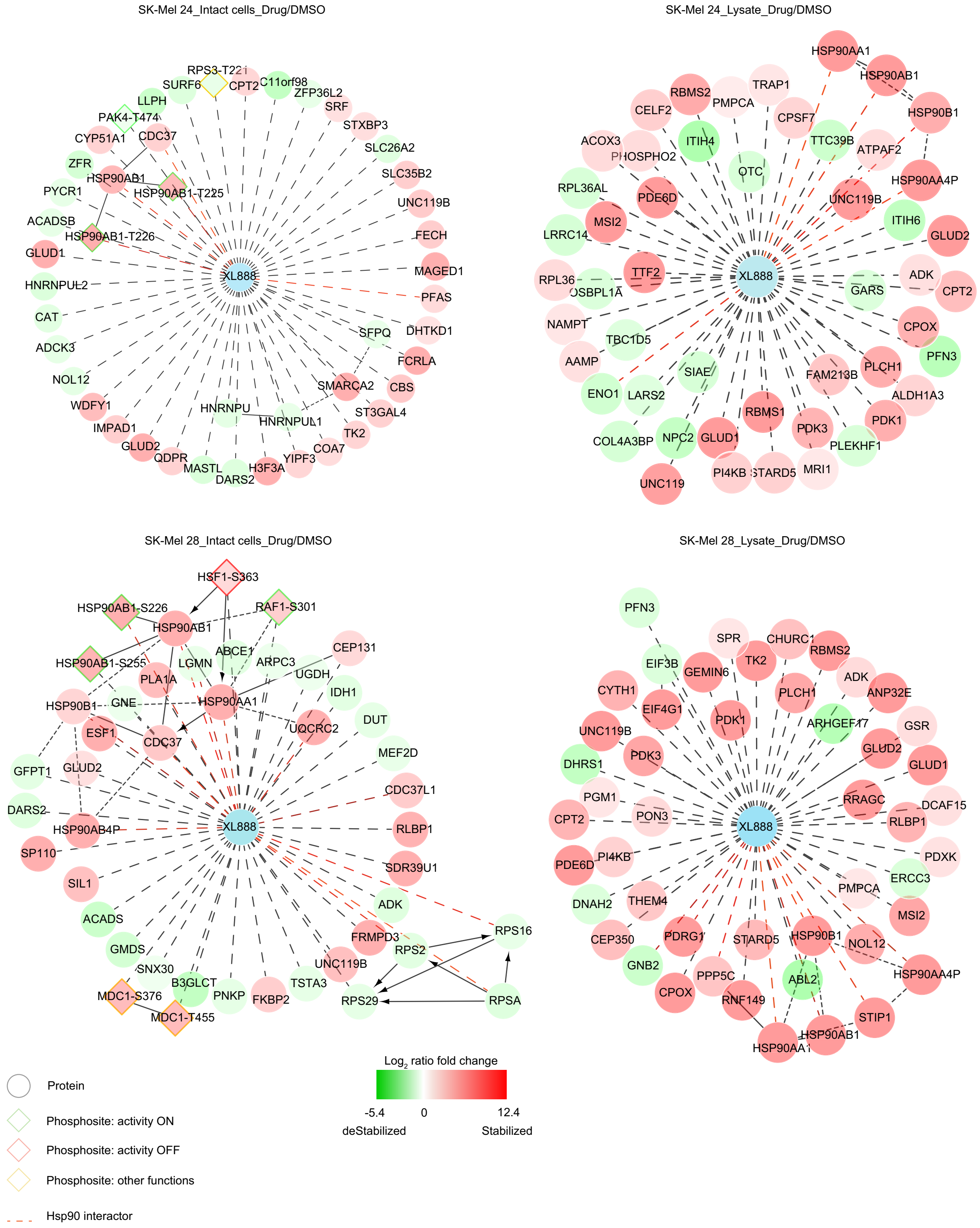

Figure EV2. TPP and phospho-TPP protein interaction maps in sensitive and resistant cells.

Protein interaction map built using Cytoscape 3.2 and Reactome as plugin (see Appendix) and the statistical significant entries generated from the comparison of the proteome and phosphoproteome of drug versus DMSO for sensitive and resistant cells in both "lysate" and "intact cell" layouts.

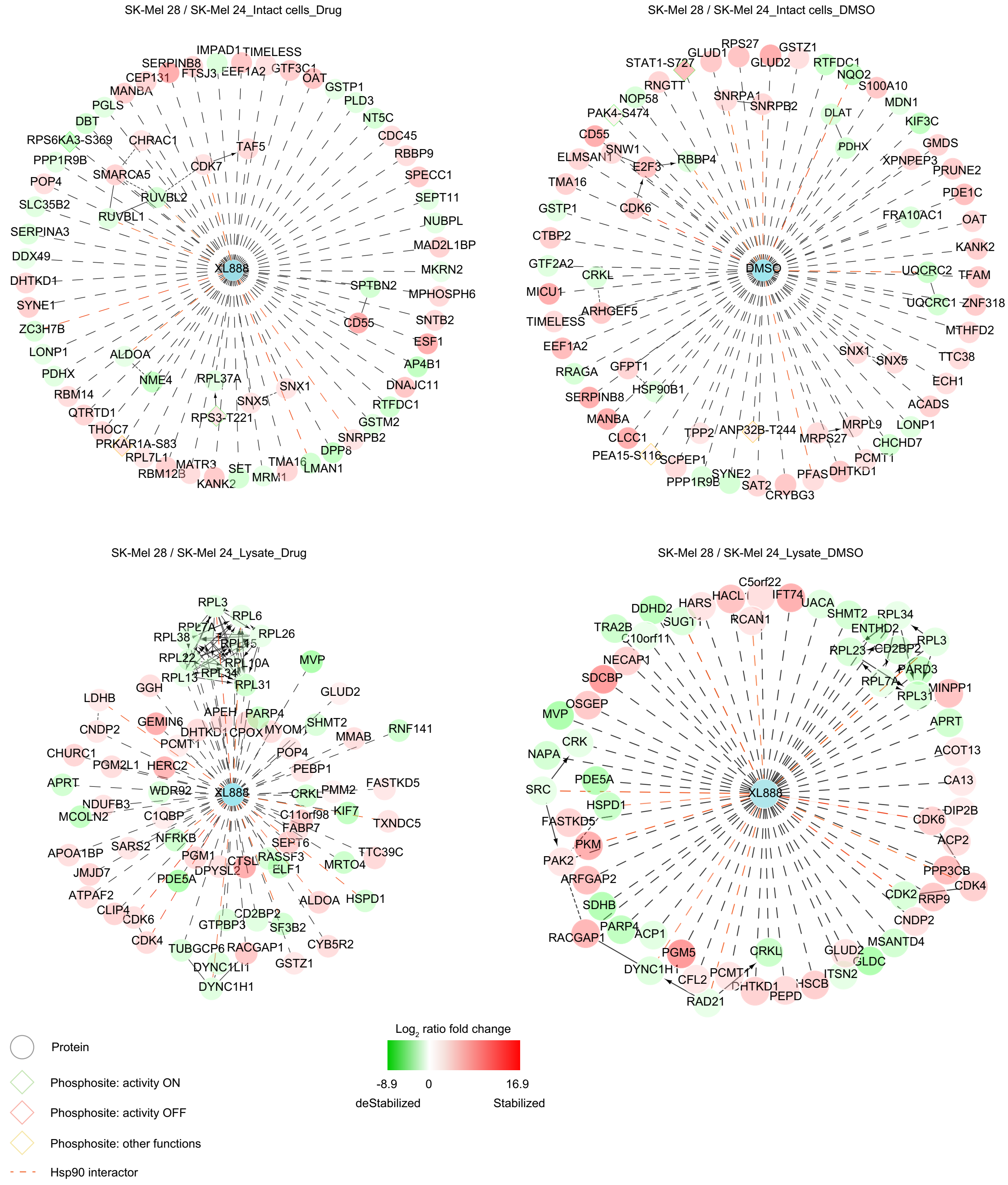

Figure EV3. Proteome thermal stability of resistant versus sensitive cells.

Protein interaction map built using Cytoscape 3.2 and Reactome as plugin (see Appendix) and the statistical significant entries generated from the comparison of the proteome and phosphoproteome thermal stability of resistant versus sensitive cells different settings.

 © 2018 The Authors

Figure EV4. Validation of the TPP and proteomics findings.

A Melting curves of some hits generated by the comparison of the baseline thermal stability of SK-Mel 24 and SK-Mel 28 in lysate and intact cell settings.
B Validation of pPAK4 thermal shift by Western blot.
C Principal component analysis (PCA) of the proteomics (left panel) and phosphoproteomics (right panel) results in different settings.
D Western blot analyses of SK-Mel 24 and SK-Mel 28 upon BRAFi treatment show phosphorylation and activation of pERK.
E Protein expression levels of the shared kinases statistically significant regulated in SK-Mel 24 and SK-Mel 28 upon treatment with BRAFi-Hsp90i/DMSO.
F Cell viability measurements ± doxycycline (72–96 h) for the non-targeting scrambled shRNA (NT CTL), CDK2, and MITF conditional knockdown cell lines in DMSO (±SD is plotted; n = 3).
G Western blots of protein expression levels of CDK2 in A375 DR1, ESTDAB 37, M026.X1.CL, and MNT-1 DR100 upon treatment with DMSO, BRAFi, and Hsp90i at 72 h (left panel). Band intensities for the quantification of CDK2 expression levels in different cell lines in different settings were normalized against the mean of GAPDH, and DMSO treatment was used as reference (right panel).
H Western blot of protein expression levels of AKT1 in SK-Mel 24 and SK-Mel 28 in different settings at 48-h treatment.

© 2018 The Authors

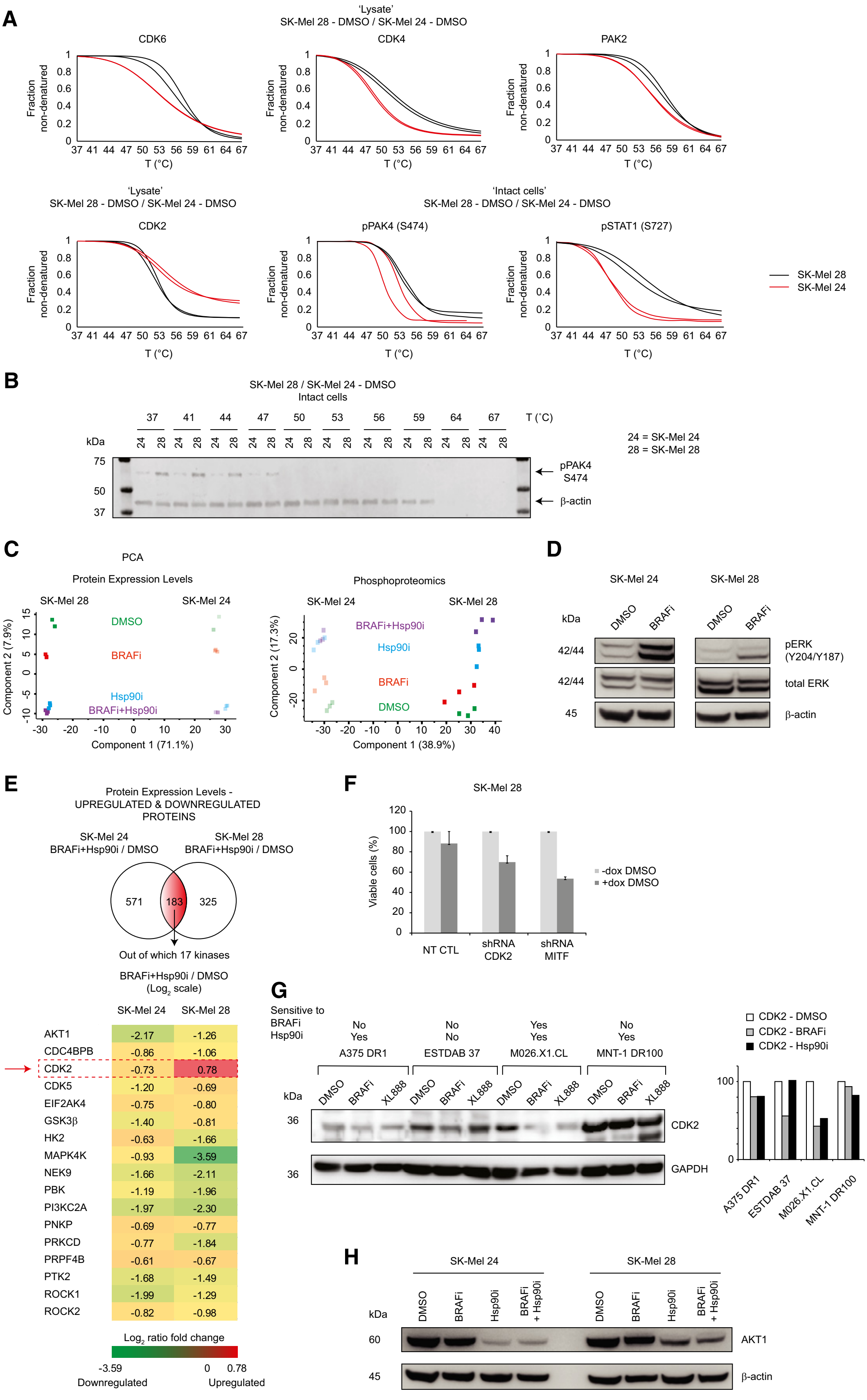

Figure EV4.

© 2018 The Authors
