# Supplementary material for: Targeting CDK2 overcomes melanoma resistance against BRAF and Hsp90 inhibitors
Source: Mol Syst Biol. 2018 Mar 5;14(3):e7858. doi: 10.15252/msb.20177858 (PMC5836539; doi:10.15252/msb.20177858)
Supplement: Supplementary file 11 — Code EV1 [file MSB-14-e7858-s011.zip › MSB_7858_Computer_Scripts_EV1/ReadMe.docx]

These files represent an in house data flow for fitting, analyzing and visualization outputs for MS-CETSA (TPP) for phospho-enriched samples and are therefore not systematically documented. The majority of the fitting procedures are adapted directly from the available TPP pipeline (PMID: 26524241, DOI: 10.1038/nmeth.3652).

Files in the pipeline should be run in the following order:

fit_phospho.R

analysis_phospho.R

print_pdf_phospho.R

and require a separate file with commands (specify location on line 66 of the fit_phospho script).

Input files for the first script are those directly coming from MaxQuant and all subsequent files and locations are specified by the user within the scripts. All scripts are written on and compatible with a PC.

This pipeline has only been used for one dataset and therefore all analysis values should be explicitly examined when adapting the procedure to further datasets. Users are referred to the papers on the TPP pipeline for in depth description of a majority of these factors (PMID: 26379230, DOI: 10.1038/nprot.2015.101; PMID: 25278616, DOI: 10.1126/science.1255784).

For all additional enquiries please contact the corresponding author.
